# Post-translational Protein Acetylation: An Elegant Mechanism for Bacteria to Dynamically Regulate Metabolic Functions

**DOI:** 10.3389/fmicb.2019.01604

**Published:** 2019-07-12

**Authors:** David G. Christensen, Xueshu Xie, Nathan Basisty, James Byrnes, Sean McSweeney, Birgit Schilling, Alan J. Wolfe

**Affiliations:** ^1^Health Sciences Division, Department of Microbiology and Immunology, Stritch School of Medicine, Loyola University Chicago, Maywood, IL, United States; ^2^Buck Institute for Research on Aging, Novato, CA, United States; ^3^Energy & Photon Sciences Directorate, National Synchrotron Light Source II, Brookhaven National Laboratory, Upton, NY, United States

**Keywords:** acetylation, lysine acetyltransferase, bacteria, mass spectrometry, proteomics

## Abstract

Post-translational modifications (PTM) decorate proteins to provide functional heterogeneity to an existing proteome. The large number of known PTMs highlights the many ways that cells can modify their proteins to respond to diverse stimuli. Recently, PTMs have begun to receive increased interest because new sensitive proteomics workflows and structural methodologies now allow researchers to obtain large-scale, in-depth and unbiased information concerning PTM type and site localization. However, few PTMs have been extensively assessed for functional consequences, leaving a large knowledge gap concerning the inner workings of the cell. Here, we review understanding of *N*-𝜀-lysine acetylation in bacteria, a PTM that was largely ignored in bacteria until a decade ago. Acetylation is a modification that can dramatically change the function of a protein through alteration of its properties, including hydrophobicity, solubility, and surface properties, all of which may influence protein conformation and interactions with substrates, cofactors and other macromolecules. Most bacteria carry genes predicted to encode the lysine acetyltransferases and lysine deacetylases that add and remove acetylations, respectively. Many bacteria also exhibit acetylation activities that do not depend on an enzyme, but instead on direct transfer of acetyl groups from the central metabolites acetyl coenzyme A or acetyl phosphate. Regardless of mechanism, most central metabolic enzymes possess lysines that are acetylated in a regulated fashion and many of these regulated sites are conserved across the spectrum of bacterial phylogeny. The interconnectedness of acetylation and central metabolism suggests that acetylation may be a response to nutrient availability or the energy status of the cell. However, this and other hypotheses related to acetylation remain untested.

## Introduction

The two competing theories for the origin of life (metabolism-first and gene-first) suggest that abiotic chemical reactions were the progenitors of biotic life. These abiotic chemical reactions ultimately formed the basis for metabolism and provided building blocks for the synthesis of both RNA and protein. Over millions of years, cells formed and evolved and, in doing so, generated the diversity of life we observe today. Across phylogeny, the core metabolic pathways necessary for life have been well conserved (Peregrín-Alvarez et al., 2009). In these core pathways, anabolism and catabolism generate a multitude of different metabolites. The potential chemical reactions in which these metabolites can participate greatly exceed cellular metabolic requirements. Therefore, the cell has evolved diverse mechanisms to control these “extra-metabolic” compounds to limit their toxicity or the damage that they impart upon macromolecular structures via non-enzymatic reactions within the cell (Dickinson and Chang, 2011). While cells have evolved means of controlling these “extra-metabolic” compounds, it is thought that accumulation of the resultant damage may be a prototypic driver of aging (Golubev et al., 2017).

Despite the constant possibility of damage due to their own metabolism, bacteria have survived and evolved to adapt to various niches. However, these environments are rarely constant outside of a laboratory setting, and bacteria will often face adverse conditions. In these niches, one of the major challenges is acquisition of nutrients necessary to grow and divide. While some nutrients like carbon may be readily abundant, other nutrients can be scarce, such as iron sequestered by a host (Hood and Skaar, 2012). These conditions may change suddenly, so the bacteria must respond accordingly or perish. It is well established that cells respond to environmental challenges with appropriate regulation of their transcriptional and translational programs (Gottesman, 2017). Whereas transcription and translation are time- and energy-intensive processes, post-translational control over the function of existing proteins can rapidly alter cellular physiology and permit fitness or survival in otherwise suboptimal or lethal conditions (Castaño-Cerezo et al., 2014; Lynch and Marinov, 2015). While small ligands may allosterically regulate proteins, diverse covalent chemical modifications of the existing proteome also occur; these may or may not be reversible (Martin, 2014). Researchers have identified hundreds of chemical modifications of the proteome; these are called post-translational modifications (PTMs). These PTMs can occur on a variety of protein residues and understanding how these modifications affect protein structure and function and whether they interact with one another is critical to generating a complete regulatory picture of the cell.

Many PTMs occur on lysines within proteins, including methylation (Lanouette et al., 2014), propionylation (Chen et al., 2007), butyrylation (Chen et al., 2007), crotonylation (Tan et al., 2011; Sabari et al., 2015), succinylation (Zhang et al., 2011), malonylation (Du et al., 2011; Peng et al., 2011), glutarylation (Tan et al., 2014), 2-hydroxyisobutyrylation (Dai et al., 2014), pupylation (Becker and Darwin, 2017), and *N*𝜀-lysine acetylation, which is becoming recognized as a prominent modification that is conserved across phylogeny (Kim et al., 2006; Choudhary et al., 2009). While lysine acetylation is best understood as an activating modification of eukaryotic histones (Hebbes et al., 1988), extensive study into the role of lysine acetylation in bacteria only began about a decade ago. Acetylation of certain lysines may have a clear consequence, such as inhibition of enzyme activity due to modification of an active site (Nakayasu et al., 2017), but the functional importance or lack thereof of many acetyllysine modifications are more difficult to discern. To uncover the role of lysine acetylation in these unclear cases, it is helpful to use a model bacterium (e.g., *Escherichia coli*) with a vast knowledgebase of pathways, protein structure-function relationships, and physiology.

This review will detail what we know concerning lysine acetylation in bacteria with some attention paid to eukaryotic acetylation for context. We will begin by describing the different types of acetylation, the mechanisms that result in acetylation of lysines, and some known effects of lysine acetylation on protein function. We will discuss the importance of mass spectrometry to identify and quantify acetylated proteins. Furthermore, we will explore the possibility that acetylation could be a driver of protein and organismal evolution. Finally, with our deepening understanding of acetylation, we will discuss a possible future for this new field.

## Acetylation – From Small Molecules to Proteins

Acetylation is simply the transfer of an acetyl group (CH_3_CO) onto a molecule. The acetyl group can react with a variety of atoms or functional groups on a target molecule. The atom to which the acetyl group is attached is usually denoted in the name of either the final molecule or the enzyme that performs the acetylation. Acetylation can occur with thiol groups (sulfur), hydroxyl groups (oxygen), and often amino groups (nitrogen).

Acetylation reactions can target small molecules and metabolites, or they can occur on proteins. Multiple metabolic pathways require the transfer of acetyl groups from one metabolite to the next. For example, *N*-acetylglutamate synthase catalyzes acetylation of L-glutamate, the first step in arginine biosynthesis (Cunin et al., 1986). Acetylation of multiple aminoglycoside antibiotics has been reported to inactivate their bactericidal or bacteriostatic activity (Kawabe et al., 1975; Shaw and Hopwood, 1976; Garneau-Tsodikova and Labby, 2016), and acetylation of the antibiotic chloramphenicol disables its ability to inhibit translation (Bulkley et al., 2010). The chitin of insects, plants, and fungi and the peptidoglycan of the bacterial cell wall are composed of complex polysaccharides (Bernard et al., 2011). These polysaccharides often contain monomers such as *N*-acetylglucosamine (GlcNAc) and *N*-acetylmuramic acid (MurNAc), which are monosaccharide derivatives acetylated on the primary amine (N), as their names suggest. However, these can be further acetylated on hydroxyl groups to increase resistance to degradative enzymes, such as lysozyme and autolysins (Bernard et al., 2011).

The most widely studied protein acetylations occur on amino groups, although acetylations on serine, threonine, and histidine residues of proteins also have been detected (Mukherjee et al., 2006, 2007; Paquette et al., 2012; Lee et al., 2015). Two distinct mechanisms for acetylation of amino groups of proteins can occur: lysine acetylation (*N*𝜀-acetylation) and N-terminal protein acetylation (*N*α-acetylation). Lysine acetylation will be discussed in more detail below. *N*α-acetylation is very common in eukaryotes, but rare in bacteria with only 47 proteins detected as N-terminally acetylated in *E. coli*, 117 identified in *Pseudomonas aeruginosa*, and 145 identified in *Acinetobacter baumannii* (Ouidir et al., 2015; Kentache et al., 2016; Brown et al., 2017). Acetylation is observed either on the free amino group of methionine or on the exposed amino acid after cleavage of the N-terminal methionine. The modification is usually co-translational in eukaryotes but post-translational in bacteria, mitochondria, and chloroplasts, as the methionine first must be deformylated (Polevoda and Sherman, 2003). *N*α-acetylation has been shown to alter protein stability in eukaryotes and is typically irreversible (Helbig et al., 2010; Helsens et al., 2011). *E. coli* encodes three proteins that have demonstrated *N*α-acetyltransferase activity: RimI, RimJ, and RimL. Interestingly, the only known targets of RimI, RimJ, and RimL are the ribosomal proteins S5, L12, and S18, respectively (Yoshikawa et al., 1987; Tanaka et al., 1989). The exact physiological effects of *N*α-acetylation in *E. coli* are not known. There is evidence that RimJ plays a role in ribosome assembly, but it is not known whether this phenotype requires the *N*α-acetyltransferase activity of RimJ (Yoshikawa et al., 1987; Roy-Chaudhuri et al., 2008). RimJ also plays a role in transcriptional regulation of the P pilus of uropathogenic *E. coli*, but the specific target and whether *N*α-acetyltransferase activity is required remain unknown (White-Ziegler and Low, 1992; White-Ziegler et al., 2002). One defined role for *N*α-acetylation comes from the ESAT-6 virulence factor of *Mycobacterium tuberculosis*. Cleavage of the N-terminal methionine of ESAT-6 reveals a threonine, which is then acetylated on the amino terminus. This acetylation alters protein–protein interactions with the binding partner CFP-10, which attenuates virulence (Okkels et al., 2004;Lange et al., 2014; Mba Medie et al., 2014).

Until recently, the only known internal residue that could be acetylated was lysine. However, recent work from a few groups found that a family of bacterial effector molecules from certain pathogenic species can acetylate serine, threonine, and histidine in addition to lysine residues. In some *Yersinia* species, YopJ can act as an acetyltransferase to modify serine and threonine residues of kinases in mammalian host cells to subvert host immunity (Mukherjee et al., 2006, 2007; Mittal et al., 2010; Paquette et al., 2012). Similarly, HopZ3, a YopJ family member from the plant pathogen *Pseudomonas syringae*, can acetylate plant proteins on lysine, serine, threonine, and histidine residues, as well as bacterial effector proteins AvrB and AvrB3 (Lee et al., 2015). In both cases, the acetylation by YopJ or HopZ3 prevents effective immune signaling, which results in successful bacterial infection. This does not appear to be an exclusively inter-kingdom modification, because *O*-acetylation of serine and threonine was found to occur on a proteome-wide scale in *M. tuberculosis* (Birhanu et al., 2017). Finally, *O*-acetylation of a single serine (S608) in acetyl-CoA synthetase (Acs) regulates the ability of a neighboring lysine (K610) to be acetylated in *Streptomyces lividans* (VanDrisse and Escalante-Semerena, 2018). The discovery of these unusual acetylated residues opens new questions concerning the prevalence of these modifications and their potential interaction with other PTMs.

## *N*𝜀-Lysine Acetylation

**FIGURE 1:**
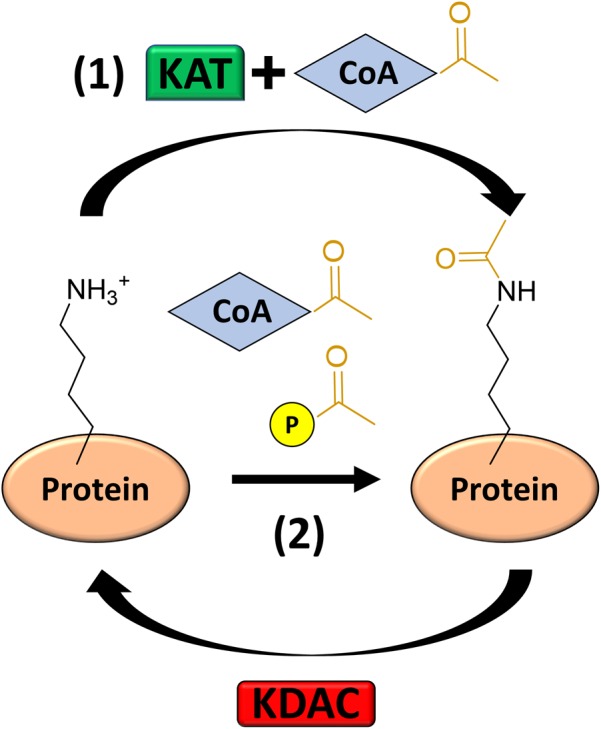
Mechanisms of Acetylation. Acetylation can be catalyzed (1) by a lysine acetyltransferase (KAT) using acetyl-CoA as the acetyl donor or (2) non-enzymatically by acetyl phosphate or acetyl-CoA. Some, but not all, acetylations can be reversed by a lysine deacetylase (KDAC).

*N*𝜀-lysine acetylation (hereafter acetylation) is the donation of an acetyl group onto the epsilon amino group of a lysine sidechain (Figure 1). Acetylation increases the size of the side chain and neutralizes the positive charge (+1 to 0) when the pH is less than the pKa of the specific lysine residue. Losing the positive charge and increasing the size of lysine disrupts salt bridges and introduces steric bulk that can alter protein–protein interactions, protein–DNA interactions, stability, and enzymatic activity (Glozak et al., 2005; Yang and Seto, 2008a; Xiong and Guan, 2012). This was first and extensively studied in the context of the histone code of eukaryotes (Phillips, 1963), where acetylation of the lysine rich C-terminal tail reduces protein–DNA binding. This remodels chromatin to activate gene transcription (Allfrey et al., 1964; Gershey et al., 1968; Vidali et al., 1968; Hebbes et al., 1988). However, recent mass spectrometric studies of acetylation have detected acetylation in all three domains of life (Zhao et al., 2003; Marsh et al., 2005; Spange et al., 2009; Zhang et al., 2009). The consortium of acetylated proteins is often called the acetylome. The first bacterial acetylome was reported in 2008 (Yu et al., 2008); since then >50 bacterial acetylomes have been reported (Christensen et al., 2019). This accumulation of bacterial acetylomes has resulted from advances in mass spectrometry-based proteomic approaches.

## Mass Spectrometry-Based Proteomics to Study Bacterial Acetylomes

Due to its high sensitivity and high-throughput, high-resolution mass spectrometry has emerged as a powerful technique to study lysine acetylation (Doll and Burlingame, 2015). For example, one can profile the global lysine acetylome of an organism, identifying acetyllysine (K^ac^) sites within a given proteome. Alternatively, one can use targeted approaches to examine specific acetyllysine sites of biological interest with high sensitivity and quantitative accuracy (Kuo and Andrews, 2013).

To compare the acetylome under different conditions, studies can be designed and mass spectrometric workflows can be optimized to not only identify K^ac^ sites, but also to detect dynamic changes in terms of relative acetylation abundance over time, in isogenic sets of mutants, or in response to different treatments. These methods have been reviewed previously (Carabetta and Cristea, 2017). Such quantitative studies can be quite impactful and relevant. For example, two studies have provided insight into the dynamic changes that occur in response to the nutritional capacity of the bacterial environment (Kuhn et al., 2014; Schilling et al., 2019). Another study characterized the acetylome of drug resistant *Salmonella* strains, identifying associations between acetylation and drug resistance (Li L. et al., 2018). A recent review described how bacterial protein acetylation might mediate bacterial virulence (Ren et al., 2017). Quantitative mass spectrometric analysis of acetylation sites also has proven helpful in identifying novel lysine acetyltransferases (KATs) (Christensen et al., 2018; Carabetta et al., 2019).

In most mass spectrometry-based workflows, such as those described above, proteins are first subjected to proteolytic digestion, and peptides are immuno-enriched with anti-K^ac^ antibodies before mass spectrometric analysis (Liu et al., 2008; Yu et al., 2008; Kuhn et al., 2014; Schilling et al., 2015, 2019; Xie et al., 2015). However, it is also possible to detect acetylation directly from bacterial whole lysates. For example, a large bacterial acetylation study was carried out by Nakayasu and co-workers to investigate the proteomes and acetylomes of 48 phylogenetically distant bacteria, identifying more than 9,000 acetylated proteins with approximately 190 acetylated proteins per organism, and more than 24,000 acetylated peptides with approximately 508 K^ac^ per organism (Nakayasu et al., 2017). Many of the observed K^ac^ sites were conserved across the phylogenetic spectrum, leading the authors to conclude that lysine acetylation is an evolutionarily conserved mechanism and that bacterial cells use it to regulate central metabolism (Nakayasu et al., 2017).

As mass spectrometric techniques and software have improved, the breadth of targets modified by acetylation has expanded. It is now clear that the extent of acetylation, the mechanisms by which acetylation occurs, the identities of acetylated proteins, and the locations of acetylation on those proteins can vary between organisms; yet, in most cases, there is great conservation in core cellular processes (Nakayasu et al., 2017). Such approaches have shown that acetylation occurs on proteins involved in a wide diversity of cellular process, especially central metabolism and translation (Wang et al., 2010; Weinert et al., 2013a; Baeza et al., 2014; Kuhn et al., 2014; Kosono et al., 2015; Schilling et al., 2015; Kentache et al., 2016; Birhanu et al., 2017; Jers et al., 2017; Nagano-Shoji et al., 2017; Post et al., 2017; Bontemps-Gallo et al., 2018; Gaviard et al., 2018; Sun et al., 2018; Vasileva et al., 2018; Yoshida et al., 2019). As stated above, many of these acetylations are conserved. This conservation has led some to hypothesize that the mechanisms of acetylation and the consequences of those acetylations are also conserved (Nakayasu et al., 2017).

## Acetyltransferases in Bacteria

Multiple families of acetyltransferases have been discovered across phylogeny. Many of these families were discovered in relation to histone acetylation in eukaryotes. Two major families of acetyltransferases are the Gcn5-related *N*-acetyltransferases (GNATs) and MYST families (Yang et al., 1996; Vetting et al., 2005). Two smaller groups of acetyltransferases known as the p300/CBP family and the SRC family also exist, as well as the unique acetyltransferases TAF_II_250, TFIIIC, Rtt109, and CLOCK (Bannister and Kouzarides, 1996; Mizzen et al., 1996; Spencer et al., 1997; Marmorstein and Roth, 2001). The kinetic mechanisms of these acetyltransferases vary. While GNATs form a ternary complex allowing a direct transfer of the acetyl group, the MYST family can either use this ternary complex mechanism or utilize a ping-pong mechanism that generates an acetylated enzyme intermediate (Yan et al., 2002). In contrast to these larger families, the p300/CBP family has been shown to use a sequential mechanism called the Theorell-Chance mechanism (Liu et al., 2008). Since GNATs are the predominant family present in bacteria, we will focus on them.

The GNATs are present across bacterial phylogeny (Neuwald and Landsman, 1997) and modify a wide range of substrates, including not only proteins, but also small molecules or metabolites (Shaw et al., 1993; Davies and Wright, 1997; Vetting et al., 2005). GNATs are generally small proteins that bind acetyl-CoA (AcCoA). They have low sequence identity, but share a characteristic fold by which they are identified (Favrot et al., 2016). The number of GNATs contained in a given genome can vary wildly with *Listeria monocytogenes* containing ∼14 GNATs, but *Streptomyces lividans* containing ∼72 GNATs (Hentchel and Escalante-Semerena, 2015).

The lysine acetylation reaction commonly occurs in a ternary complex between the substrate, AcCoA, and the GNAT, or more specifically KAT. There are three key steps to this acetylation reaction: (1) deprotonation of the target lysine, (2) nucleophilic attack of the lysine on the carbonyl carbon of the acetyl group of AcCoA, and (3) protonation and dissociation of CoA (Vetting et al., 2005). Within the catalytic pocket of the KAT, there is often a catalytic glutamate that acts as a general base to deprotonate the target lysine. In the namesake Gcn5 enzyme of yeast, this glutamate is E173, which can be found conserved in many of the bacterial GNATs (Tanner et al., 1999; Trievel et al., 1999; Liszczak et al., 2011) and some MYST family members in eukaryotes (Yan et al., 2002; Berndsen et al., 2007). However, in the absence of this conserved glutamate, it is hypothesized that a series of residues form a water channel or “proton wire” whereby protons are carried away from active site to other residues or to the solvent (Dyda et al., 2000). Once the lysine is deprotonated, it can perform a nucleophilic attack on the carbonyl carbon of AcCoA, which is bound to the KAT via a positively charged patch that coordinates the CoA moiety via its pyrophosphate and pantothenate moieties (Dutnall et al., 1998; Lin et al., 1999; Tanner et al., 1999; Marmorstein and Roth, 2001; Liu et al., 2008). The newly acetylated substrate dissociates from the KAT, but for the CoA to dissociate, it must first be protonated. Often, a tyrosine in the active site donates a proton to the thiolate of CoA.

In bacteria, GNATs were first characterized as aminoglycoside *N*-acetyltransferases (Wright and Ladak, 1997; Wolf et al., 1998). Except those that belong to the YopJ effector protein superfamily (Lewis et al., 2011; Ma and Ma, 2016), currently every KAT discovered in bacteria belongs to the GNAT family (Latrasse et al., 2008; Thao and Escalante-Semerena, 2011; Dancy and Cole, 2015). In *E. coli*, five KATs have been identified with YfiQ (also called Pat, PatZ, or Pka) being the most heavily studied (Wang et al., 2010). Homologs of YfiQ exist in many bacterial species, as described below, but additional KATs with distinctly different structures have been identified, including homologs of the other KATs in *E. coli*: RimI, YiaC, YjaB, and PhnO (Christensen et al., 2018).

## Deacetylases in Bacteria

To make a PTM versatile and energetically efficient, the ability to add and remove the PTM is essential. Lysine acetylation can be reversed via the action of a lysine deacetylase (KDAC). Two types of deacetylases are known: NAD^+^-dependent sirtuins (Blander and Guarente, 2004) and Zn^2+^-dependent deacetylases (Marmorstein, 2001; Gregoretti et al., 2004; Yang and Seto, 2008b; Lombardi et al., 2011). Putative homologs of both families are encoded by bacteria, but only a few have been shown to function as true deacetylases (Hildmann et al., 2007; Hentchel and Escalante-Semerena, 2015).

The Zn^2+^-dependent deacetylases are simple hydrolases that cleave the acetyl group from the lysine to release acetate. The reaction requires that a conserved histidine residue act as a general base to activate a metal-bound water that attacks the carbonyl of the acetyl group. This class of deacetylase has yet to be found in *E. coli*, but AcuC of *Bacillus subtilis* (Gardner et al., 2006) and LdaA of *Rhodopseudomonas palustris* (Crosby et al., 2010) are family members. Enzymes of the hydrolase family can be inhibited by the addition of butyrate (Davie, 2003).

Sirtuins bind NAD^+^ through a Rossmann fold domain (Sanders et al., 2010) and use the NAD^+^ as a co-substrate to remove acetyl groups from proteins; the products are then deacetylated lysine, nicotinamide (NAM) and 2′-*O*-acetyl-ADP-ribose. Since NAD^+^ levels are linked to nutritional status of the cell, it is thought that sirtuins allow the cell to recognize these changes and adjust acetylation accordingly (Wimpenny and Firth, 1972; Frye, 2000). Furthermore, sirtuins may regulate their own activity by producing NAM. NAM inhibits non-competitively by condensing with an ADP-ribose-like intermediate formed during the deacetylation reaction; this condensation blocks the progression of the reaction (Denu, 2003) at physiological levels (Wimpenny and Firth, 1972; Bitterman et al., 2002; Jackson et al., 2003; Sauve and Schramm, 2003; Zhao et al., 2004; Avalos et al., 2005). NADH also competitively inhibits the reaction at physiological levels (Schmidt et al., 2004; Guarente, 2005; Porcu and Chiarugi, 2005).

In many members of the *Enterobacteriaceae*, the sirtuin CobB exists as two isoforms. In *Salmonella enterica* and *E. coli*, a shorter isoform (CobB_S_) and the longer isoform (CobB_L_) are transcribed from two different transcription start sites (Tucker and Escalante-Semerena, 2010; Umehara et al., 2018). These isoforms differ by an N-terminal amphipathic stretch of 37 amino acids. Though the purpose of this N-terminal extension is unknown, both forms of the CobB protein were shown to be biologically active as deacetylases in *S. enterica* (Tucker and Escalante-Semerena, 2010).

While sirtuins are classically thought of as deacetylases, it appears that they may have a broader specificity than originally thought. Sirtuins in the mitochondria have been reported to desuccinylate (Du et al., 2011; Colak et al., 2013), demalonylate (Du et al., 2011; Peng et al., 2011; Park et al., 2013), dipropionate (Smith and Denu, 2007), deglutarylate (Tan et al., 2014), decrotonylate (Bao et al., 2014), and debutyrylate (Smith and Denu, 2007) lysines in addition to deacetylate. Certain human sirtuins, such as SIRT6, have a preference for removal of long-chain acyl groups from lysines. These long-chain acyl groups can be a long as 16 carbons, as in the case of palmitoylation (Feldman et al., 2013). Sirtuins of archaeon *Archaeoglobus fulgidus* have similarly been shown to desuccinylate and demyristoylate peptides (Ringel et al., 2014). Bacterial sirtuins have similar abilities to desuccinylate (Colak et al., 2013), dipropionate (Garrity et al., 2007; Sun et al., 2016b), and possibly debutyrylate (Xu et al., 2018a) lysines. Many of these PTMs occur on the same lysine, possibly implicating sirtuins as a general cleanup enzyme for certain lysines. Further complicating the possible role of sirtuins, there is evidence that sirtuins can ADP-ribosylate proteins using NAD^+^ as a substrate (Frye, 1999).

## Effects of Enzymatic Acetylation/Deacetylation on Physiology and Pathogenesis

Several studies have explored the role of enzymatic acetylation on bacterial physiology, often through reverse genetics approaches where the deacetylase, acetyltransferase, or both are deleted or overexpressed. In this section, we will focus on CobB and YfiQ of *E. coli* and their homologs in *S. enterica*, as they have been studied the most extensively.

The earliest and thus most comprehensively understood roles for YfiQ and CobB are their opposing effects on the acetylation status of Acs, an enzyme that irreversibly converts acetate into AcCoA via two half reactions. Acs requires lysine 609 (K609) for the first half-reaction, which forms an acetyl-AMP intermediate (Starai et al., 2002). Thus, mutation or modification of this residue leads to a catalytically inactive enzyme (Starai et al., 2002; Starai and Escalante-Semerena, 2004a,b; Sun et al., 2016b). Indeed, an epistasis analysis of Δ*acs*, Δ*cobB*, and Δ*yfiQ* mutants showed that regulation of Acs is essential for growth on acetate. In both *S. enterica* and *E. coli*, Δ*acs* mutants cannot grow on less than 10 mM acetate as the sole carbon source. This is due to the low affinity of the alternative Pta-AckA pathway for acetate. A Δ*cobB* mutant phenocopies a Δ*acs* mutant, while the double Δ*yfiQ*Δ*cobB* or Δ*pat*Δ*cobB* mutants can grow on less than 10 mM acetate (Starai et al., 2003; Castaño-Cerezo et al., 2011). These results confirm that YfiQ/Pat-dependent acetylation of Acs shuts down acetate utilization through Acs and that deacetylation by CobB restores that activity (Starai et al., 2002; Castaño-Cerezo et al., 2011). Acs was found to be regulated in the exact same fashion in more than 15 organisms tested, ranging from bacteria to humans (Table 1) (Kim and Yang, 2011). Beyond Acs, this reversible lysine acetylation has been extended to other AMP-forming acyl-CoA synthetases, which are proteins that share a similar structure and the same conserved acetylated lysine found in Acs. *R. palustris* possesses 40 of these Acs-like proteins; 10 of them are reversibly acetylated on this conserved lysine (Crosby et al., 2010, 2012). As in Acs, acetylation inactivates these enzymes.

**Table 1:** Proteins assessed for regulation by acetylation.

Organism	Target	Target lysine(s)	Acetylation	Deacetylation	Acetylation	Cite
	protein		mechanism	mechanism	effect	
*Actinosynnema mirum*	Acs	620	*Ami*PatA and 23 homologs	Not determined	Inhibits activity	Lu et al., 2017
*Bacillus subtilis*	Acs	549	AcuA	AcuC, SrtN	Abolishes activity	Gardner et al., 2006; Gardner and Escalante-Semerena, 2008, 2009
	Eno	339, 390	*In vitro* AcP	Not determined	Abolishes activity	Nakayasu et al., 2017
	HBsu	3, 18, 41, 80	YfmK	Not determined	Decreases DNA binding	Carabetta et al., 2019
	TufA	42	Not AcuA, not AcP	AcuC, SrtN	No effect	Suzuki et al., 2019
	MreB	240	Not determined	Not determined	Reduces cell length, width, and peptidoglycan thickness	Carabetta et al., 2016
	CshA	244, 296	Unknown	Not determined	Enhances induction of SigX and SigM-dependent genes	Ogura and Asai, 2016; Ogura and Kanesaki, 2018
*Borrelia burgdorferi*	GapA	Not determined	AcP	Not determined	Inhibits activity	Bontemps-Gallo et al., 2018
	LDH	Not determined	AcP	Not determined	Mixed results	Bontemps-Gallo et al., 2018
*Corynebacterium glutamicum*	PEPC	653	Not determined	NCgl0616	Inhibits activity	Nagano-Shoji et al., 2017
*Escherichia coli*	ArgRS	126, 408	AcP	CobB	Impairs tRNA charging	Ye et al., 2017
	CRP	100	AcP	Not determined	Reduces interaction with RNAP, enhances protein stability	Davis et al., 2018
	LeuRS	619, 624, 809	AcP	CobB	Impairs tRNA charging	Ye et al., 2017
	Mdh	99, 140	AcP	CobB for 140, none for 99	Cooperatively increases activity	Fan et al., 2015; Venkat et al., 2017b
	RpoA	291	AcP	Not determined	Reduces *cpxP* transcription	Lima et al., 2012
	TyrRS	85, 235, 238	AcP	CobB	Impairs tRNA charging	Venkat et al., 2017a
	RcsB	154	AcP	CobB	Enhances migration, impairs acid survival	Hu et al., 2013; Castaño-Cerezo et al., 2014
	TopA	13, 45, 346, 488	AcP	CobB	Reduces relaxation activity by inhibiting DNA binding and cleavage activity	Zhou et al., 2017, 2018
	DnaA	178	AcP, YfiQ	CobB	Inhibits ATP binding	Zhang et al., 2016
	DnaA	243	AcP, YfiQ	CobB	Inhibits *oriC* binding at low affinity sites	Li S. et al., 2017
	CheY	92, 109	Acs, AcCoA	Acs, Pta, CobB	Inhibits interaction with CheA, FliM, and CheZ Activates CheY, promotes clockwise rotation	Barak and Eisenbach, 2001; Barak et al., 2006; Li R. et al., 2010; Liarzi et al., 2010
	YfiQ	146, 149, 391, 446, 635, 819	AcCoA	CobB	Favors formation of octamers	de Diego Puente et al., 2015
	Eno	342, 393	*In vitro* AcP	Not determined	Abolishes activity	Nakayasu et al., 2017
	AceA	308	Not determined	CobB	Inhibits activity	Castaño-Cerezo et al., 2014
	AlaRS	73	Not determined	CobB	Inhibits activity	Umehara et al., 2018
	Mat	12 lysines	Not determined	CobB (9 lysines susceptible)	Inhibits activity, may affect dimerization	Sun et al., 2016a
	NhoA	214, 281	Not determined	CobB	Inhibits activity	Zhang et al., 2013
	RcsB	180	*Se*Pat, *Ec*YfiQ	*Se*CobB, *Ec*CobB	Inhibits DNA binding	Thao et al., 2010
	Acs	609	YfiQ	CobB	Inhibits activity by preventing first half reaction	Zhao et al., 2004; Castaño-Cerezo et al., 2011, 2014;
	protein		mechanism	mechanism	effect	
	RNase II	501	YfiQ	CobB	Inhibits RNase II activity due to reduced substrate binding	Song et al., 2016
	RNase R	544	YfiQ	NONE – not CobB	Destabilizes protein	Liang et al., 2011; Liang and Deutscher, 2012
	RpoA	298	YfiQ	Not determined	Enhances *cpxP* transcription	Lima et al., 2011
*Micromonospora aurantiaca*	Acs	619	MaKat	Not determined	Inhibits activity	Xu et al., 2014
*Mycobacterium bovis BCG*	Acs	616	KATbcg (BCG_1055)	Not determined	Abolishes activity	Nambi et al., 2013
	FadD13	487	KATbcg (BCG_1055)	Not determined	Inhibits activity	Nambi et al., 2013
*Mycobacterium smegmatis*	FadD33	511	*MsPat*	MSMEG_5175	Abolishes activity	Vergnolle et al., 2013
	MbtA	546	*Ms*Pat	Rv1151c	Inhibits activity	Vergnolle et al., 2016
	PrpE	586	*MsPat*	Not determined	Abolishes activity	Xu et al., 2018b
	MSMEG_4207 (USP)	104	*Mt*PatA, *Ms*PatA	Not determined	Unknown	Nambi et al., 2010
	Ku	29, 40	Not determined	Not determined	Correlates with impaired NHEJ activity	Zhou et al., 2015
	HupB	86	Not determined	Not determined	Prevents small colony variant formation	Sakatos et al., 2018
	Acs	589	PatA	SrtN	Impairs growth on acetate	Hayden et al., 2013
*Mycobacterium tuberculosis*	DUSP16 (eukaryotic)	55	Eis	Not determined	Inhibits activation of JNK pathway	Kim et al., 2012
	Histone H3 (eukaryotic)	Not determined	Eis	Not determined	Enhances binding to the IL-10 promoter	Duan et al., 2016
	HupB	32	Eis	Rv1151c	Inhibits DNA binding	Ghosh et al., 2016; Green et al., 2018
	Acs	617	*Ms*Pat	Rv1151c, MSMEG_5175	Abolishes activity	Xu et al., 2011; Nambi et al., 2013; Noy et al., 2014
	MbtA	542	*Ms*Pat	Rv1151c	Inhibits activity	Vergnolle et al., 2016
	DosR	182	*Mt*Pat	Rv1151c	Inhibits DNA binding	Bi et al., 2018; Yang et al., 2018
	FadD2, FadD4, FadD5, FadD10, FadD12, FadD13, FadD22, FadD35	551, 525, 519, 519, 523, 487, 480, 529	*Mt*Pat	MSMEG_5175	Impairs palmitoyl-AMP synthesis activity for FadD2, FadD5, and FadD15	Nambi et al., 2013
	PtpB	224	*Mt*Pat	Rv1151c	Decreases reaction rate due to reduced Vmax	Singhal et al., 2015
	HspX	64, 78, 85	Not determined	Not determined	Reduced immunogenicity	Liu et al., 2014
	ICL1 (Rv0467)	322 331 392	Not determined	Not determined	Inhibits activity (K322) Enhances activity (K331) Enhances activity (K392)	Xie et al., 2015; Bi et al., 2017
	Histone H3 (eukaryotic)	9, 14	Rv3423.1	Not determined	Inhibits DNA binding by histone H3	Jose et al., 2016
*Myxococcus xanthus*	Acs	622	*Mx*Kat	Not determined	Inhibits activity	Liu et al., 2015
*Neisseria gonorrhoeae*	PilT	117	AcP	Not determined	May contribute to membrane association,	Hockenberry et al., 2018
	protein		mechanism	mechanism	effect	
					alters microcolony formation	
*Porphyromonas gingivalis*	RprY	Not determined	*Pg*Pat	CobB	Inhibits DNA binding	Li Y. et al., 2018
	pro-RgpB	247, 248	VimA, PG1842	Not determined	Permits proper processing of pro-RgpB	Mishra et al., 2018
*Rhodobacter sphaeroides*	FnrL	175, 213, 223	AcP	*Rs*CobB	Impairs transcriptional activation	Wei et al., 2017
*Rhodopseudomonas palustris*	FadD	546	*Rp*Pat	Not determined	Inhibits activity	Crosby et al., 2012
	HcsA	524	*Rp*Pat	LdaA	Inhibits activity	Crosby et al., 2012
	LcsA	499	*Rp*Pat	LdaA	Inhibits activity	Crosby et al., 2012
	PimA	534	*Rp*Pat	Not determined	Inhibits activity	Crosby and Escalante-Semerena, 2014
	Acs	606	*Rp*Pat, KatA	SrtN and LdaA	Inhibits activity	Crosby et al., 2010, 2012
	AliA	532	*Rp*Pat, KatA	SrtN and LdaA	Inhibits activity	Crosby et al., 2010, 2012
	BadA	512	*Rp*Pat, KatA	SrtN and LdaA	Inhibits activity	Crosby et al., 2010, 2012
	FcsA	496	*Rp*Pat, KatA	LdaA	Inhibits activity	Crosby et al., 2012
	HbaA	503	*Rp*Pat, KatA	SrtN and LdaA	Inhibits activity	Crosby et al., 2010, 2012
	IbuA	539	*Rp*Pat, KatA	LdaA	Inhibits activity	Crosby et al., 2012
	PrpE	598	*Rp*Pat, KatA	LdaA	Inhibits activity	Crosby et al., 2012
*Saccharomyces cerevisiae*	Pck1p	514	Esa1	Sir2	Enhances activity	Lin et al., 2009
*Saccharopolyspora erythraea*	AcsA2	611	AcP	Not determined	Inhibits activity by reducing affinity for acetate	You et al., 2014
	AcsA1, AcsA2, AcsA3	620, 628, 615	AcuA	SrtN	Inhibits activity	You et al., 2014, 2017
	GlnA1	179, 357	AcuA	SrtN	Enhances GlnA1 interaction with GlnR, which enhances DNA binding affinity	You et al., 2016
	GlnA4	319	AcuA	SrtN	Inhibits activity	You et al., 2016
*Salmonella enterica*	AceA	308	Pat	CobB	Inhibits activity	Wang et al., 2010; Crosby et al., 2012
	AceK	72, 83, 553	Pat	CobB	Enhances activation of ICDH	Wang et al., 2010; Crosby et al., 2012
	GapA	331	Pat	CobB	Enhances glycolytic activity, decreases gluconeogenesis activity	Wang et al., 2010; Crosby et al., 2012
	HilD	297	Pat	Not CobB	Enhances stability, inhibits DNA binding	Sang et al., 2016, 2017
	PhoP	201	Pat	CobB	Inhibits DNA binding	Ren et al., 2016
	PrpE	592	Pat	CobB	Abolishes activity	Garrity et al., 2007
	Lrp	36	Pat	CobB	Inhibits DNA binding	Qin et al., 2016
	Acs	609	Pat	CobB	Inhibits activity by preventing first half reaction	Starai et al., 2002, 2005; Starai and Escalante-Semerena, 2004b
	Charged aminoacyl tRNAs	Amino terminus	TacT	N/A	Inhibits translation	Cheverton et al., 2016; VanDrisse et al., 2017
	protein		mechanism	mechanism	effect	
	TacA	44	TacT	CobB	Enhances TacT activity	VanDrisse et al., 2017
*Streptomyces coelicolor*	Acs	610	Not determined	CobB1	Inhibits activity	Mikulik et al., 2012
	GlnR	142, 153, 159, 200	Not determined	CobB2	Alters DNA binding	Amin et al., 2016
*Streptomyces griseus*	StrM	70	SGR1683, *Ec*YfiQ	Not determined	Inhibits activity	Ishigaki et al., 2017
*Streptomyces lividans*	Aacs	617	*Sl*Pat	*Se*CobB	Inhibits activity	Tucker and Escalante-Semerena, 2013
	Acs	610	*Sl*PatA, *Sl*PatB	*Sl*SrtA	Inhibits activity	Tucker and Escalante-Semerena, 2013; VanDrisse and Escalante-Semerena, 2018
*Streptomyces venezuelae*	Acs	665	*Sve*PatA	Not determined	Inhibits activity	Lu et al., 2017
*Sulfolobus solfataricus*	Alba	16	Pat	Sir2	Inhibits DNA binding	Marsh et al., 2005
*Thermus thermophilus*	IPMS	332	AcCoA	TT_C0104	Inhibits activity	Yoshida et al., 2019
*Vibrio cholerae*	Acs	609	YfiQ	CobB	Inhibits activity	Liimatta et al., 2018
*Yersinia pestis*	PhoP	Not determined	YfiQ	CobB	Not determined	Liu et al., 2018

Other proteins have been shown to be targets of both YfiQ and CobB. For example, K501 of RNase II from *E. coli* is a target of YfiQ and CobB (Song et al., 2016). Acetylation of K501 by YfiQ inhibits substrate binding, which results in reduced exoribonuclease activity. Treatment of RNase II with CobB restores this activity. YfiQ and CobB also reversibly acetylate K201 of PhoP from *S. enterica*. A member of a two-component regulatory system that responds to low Mg^2+^ and acid stress, Pat-dependent acetylation of K201 decreases the affinity of PhoP for its DNA binding site and thus reduces expression of PhoP-regulated genes (Ren et al., 2015, 2016). CobB restores DNA binding by deacetylating PhoP. Finally, the highly conserved global transcriptional regulator Lrp (Brinkman et al., 2003; Martínez-Antonio and Collado-Vides, 2003) has been shown to be acetylated on K36 in *S. enterica*, which prevents DNA binding (Qin et al., 2016). CobB treatment permits DNA binding of Lrp.

While the mechanistic role of reversible lysine acetylation on Acs and a few other proteins is established, multiple phenotypes have been found for Δ*pat* and Δ*cobB* mutants in *S. enterica* that are not directly attributed to acetylation of a specific enzyme, but are consistent with the opposing effects of these enzymes. For example, compared to wild-type cells, a Δ*pat* mutant of *S. enterica* grows more slowly on minimal glucose but more rapidly on minimal citrate, whereas a Δ*cobB* mutant has the exact opposite phenotype (Wang et al., 2010). The Δ*pat* mutant has reduced flux through glycolysis and the TCA cycle, while a Δ*cobB* mutant again has the opposite phenotype and promotes gluconeogenesis (Wang et al., 2010). For the latter phenotype, a Δ*cobB*Δ*pat* double mutant behaves like a Δ*pat* mutant, suggesting that CobB is dispensable in the absence of acetylation via Pat, as expected if the role of CobB was simply to deacetylate Pat-dependent acetylations. Taken together, these results support the hypothesis that Pat and CobB somehow regulate central metabolism. However, the Pat- and CobB-sensitive lysines responsible for these behaviors are not known. Acetylation by Pat was reported on isocitrate lyase (AceA), isocitrate dehydrogenase (Icd), and isocitrate dehydrogenase kinase/phosphatase (AceK), and this acetylation was shown to reduce activity for each protein (Wang et al., 2010); however, these results were called into question when another group could not confirm these findings (Crosby et al., 2012).

YfiQ/Pat, CobB, and their homologs also appear to be involved in certain stress responses. In contrast to the metabolic examples above, some of these effects might occur by modifying different lysines, as deletion of either enzyme sometimes does not result in opposite phenotypes. For example, when exposed to acid stress, both *E. coli* and *Yersinia pestis* have reduced survival when either *yfiQ* or *cobB* are deleted; in contrast, *S. enterica* survives better when *pat* is deleted and more poorly when *cobB* is deleted (Castaño-Cerezo et al., 2014; Ren et al., 2015; Liu et al., 2018). When exposed to heat stress, survival of *E. coli* diminishes when *yfiQ* is deleted but increases when *cobB* is deleted (Ma and Wood, 2011). In contrast, *Y. pestis* survival is diminished in both Δ*cobB* and Δ*yfiQ* mutants when exposed to heat, cold, or high salt stress (Liu et al., 2018). When exposed to reactive oxygen species, the *E. coli* Δ*cobB* mutant has enhanced survival, whereas the Δ*yfiQ* mutant behaves like its wild-type parent. In a more distantly related organism, *M. tuberculosis*, loss of its sirtuin (Rv1151c) promotes growth in acidic pH (Bi et al., 2017). Finally, the acetyltransferase PatA in *Mycobacterium smegmatis* acetylates the Universal Stress Protein (MSMEG_4207) (Nambi et al., 2010). Taken together, the results of these studies suggest a common role for acetylation in stress responses. The pleiotropic phenotypes of Δ*yfiQ/pat* and Δ*cobB* mutants provide evidence that the lysines they acetylate and deacetylate control a diverse set of general stress pathways.

The Regulator of Capsule Synthesis B (RcsB) can be reversibly acetylated through YfiQ and CobB *in vitro*. RcsB, a member of the two-component response regulator family, is a global transcriptional regulator of *E. coli* and *S. enterica*, regulating approximately 5 and 20% of their respective genomes (Prüss et al., 2006; Wang et al., 2007). RcsB promotes capsule biosynthesis and biofilm formation, while impairing transcription of genes required for motility. In *E. coli* and *S. enterica*, RcsB is acetylated on multiple residues. One of those lysines (K180) can be acetylated *in vitro* in a YfiQ/Pat-dependent manner and deacetylated by CobB (Thao et al., 2010). K180 is found in the RcsB DNA binding motif, and acetylation of this residue reduces DNA binding, which can then be reversed by CobB. However, there is no evidence to support YfiQ/Pat as the cause of K180 acetylation *in vivo*, as mutants that delete these KATs have no obvious motility defect (Hu et al., 2013). In contrast, two independent studies found CobB to be an inhibitor of migration in *E. coli* (Castaño-Cerezo et al., 2014; Gallego-Jara et al., 2017). A Δ*cobB* mutant has more numerous and longer flagella than the parent strain, which could partially be explained by the ability of CobB to deacetylate RcsB (Gallego-Jara et al., 2017).

Pat has been strongly implicated in *S. enterica* survival and infection. Pat enhances invasion of the bacteria into HeLa cells and enhances survival of the bacteria within macrophages (Sang et al., 2016). Pat also helps promote survival under acid stress, which may be partially responsible for the macrophage survival phenotype (Ren et al., 2015). Importantly, expression of the *Salmonella pathogenicity island* (SPI-1) that is required for virulence is enhanced by Pat (Sang et al., 2016). SPI-1 is controlled by transcription factor HilD, and acetylation on K297 of HilD by Pat was suggested to stabilize the protein, which could enhance SPI-1 transcription. The role of Pat in virulence was further established by infecting mice with Δ*pat* and Δ*cobB* mutants. The Δ*pat* mutants were hypovirulent and caused reduced mortality, implicating Pat and therefore acetylation as necessary for infection (Sang et al., 2016). However, Δ*cobB* mutants had little effect. The effect by Pat might be due, at least in part, to its ability to acetylate of K201 of PhoP, as a K201Q variant that mimics the constitutively acetylated isoform were hypovirulent (Ren et al., 2016). Like *S. enterica*, a Δ*yfiQ* mutant of *Yersinia pestis*, the causative agent of plague, is hypovirulent in mice (Liu et al., 2018), but unlike *S. enterica*, the Δ*cobB* mutant is equally attenuated.

The above examples demonstrate how mechanistically similar machinery has evolved in different organisms to suit their specific niches. In many cases, YfiQ/Pat and CobB act antagonistically to reversibly acetylate a specific lysine. However, in response to certain stresses, loss of either YfiQ/Pat or CobB can be detrimental to the survival of the bacterium. The pathogenicity data from *S. enterica* and *Y. pestis* suggest that, while some commonalities may exist between the requirements for acetylation in certain scenarios, there are many cases where YfiQ/Pat and CobB do not always act in opposition. These observations provide strong evidence that these enzymes can and do act independently and should not be considered a system.

## Non-Enzymatic Acetylation Via AcP and AcCoA

Whereas KAT-catalyzed acetylation has been known for some time, evidence has accumulated showing that non-enzymatic acetylation is also possible. *In vitro*, AcCoA and the bacterial metabolite acetyl phosphate (AcP) can non-enzymatically acetylate lysine residues on many proteins. *In vivo*, the impact that AcP has on the acetylome has been assessed in multiple bacteria. In the cases of *E. coli* (Weinert et al., 2013a; Kuhn et al., 2014), *B. subtilis* (Kosono et al., 2015; Suzuki et al., 2019), and *Neisseria gonorrhoeae* (Post et al., 2017), AcP is the predominant acetyl donor.

Chemical reactions are the basis of all life. While enzymatic catalysis is a primary driver in the kinds of substrates used and products formed, the possible chemical reactions of those substrates and products greatly exceeds those that are catalyzed (Golubev et al., 2017). Many of these uncatalyzed reactions are thought to be deleterious to the cell, causing macromolecular damage, such as oxidation of DNA or proteins. Often, the damage can be caused by products of the cell’s own metabolism, such as reactive oxygen species (Madian and Regnier, 2010). Indeed, non-enzymatic modification has been implicated in multiple human diseases (Brownlee, 2005). As cells have evolved alongside this damaging potential of their own metabolism, it is not surprising that repair pathways exist to mitigate this damage. Perhaps cells have evolved ways to utilize these extra-metabolic occurrences as means to regulate their cellular processes. Specifically, in this context, we will consider acetylation via two acetyl donors, AcCoA and AcP.

The ability of AcCoA and AcP to non-enzymatically modify proteins is not a new idea. AcP has been shown to chemically acetylate amine, thiol, and hydroxyl groups (Di Sabato and Jencks, 1961; Jencks, 1969). In the 1970s, it was discovered that AcP could non-enzymatically acetylate histones, albumin, and polylysine (Ramponi et al., 1975). Due to its high energy nature (ΔG° of -43.1 kJ/mol), which is even greater than that of AcCoA (ΔG° of -31.4 kJ/mol), AcP readily hydrolyzes in water and can acetylate without the need for an enzyme (Koshland, 1952; Voet and Voet, 1990). Furthermore, the concentrations needed to achieve non-enzymatic acetylation *in vitro* are comparable to or below physiological levels, which in *E. coli* are around 300–500 μM for AcCoA and between 200 μM and 3 mM for AcP (Vallari and Rock, 1985; Klein et al., 2007; Ramos-Montañez et al., 2010). *In vitro* acetylation reactions are often carried out at basic pH. Basic pH promotes deacetylation of lysine, which may prime the lysine for acetylation, but also increases the rate at which AcP and AcCoA dissociate (Koshland, 1952; Di Sabato and Jencks, 1961). Susceptibility to non-enzymatic acetylation differs for each lysine and each acetyl donor used. Using glutamate dehydrogenase (GDH) as an example, it was shown a single lysine (e.g., K503) could be acetylated at different rates using two different acetyl donors, AcP and AcCoA. When comparing acetylation by a single acetyl donor, multiple lysines (e.g., K110, K415, and K503) on GDH were acetylated at different rates from one another. These *in vitro* acetylation reactions with AcP and AcCoA showed that acetylation rates of these specific lysines can vary by three orders of magnitude. Furthermore, some lysines were completely non-reactive under these conditions. These findings suggest that regulation of acetylation depends on the environmental context and thus reactivity of the lysine (Baeza et al., 2015).

Assessment of non-enzymatic acetylation *in vivo* by AcCoA is difficult. In most cases, it is an essential molecule that if depleted would result in lack of viability. However, attempts to recreate the mitochondrial environment have shown that AcCoA could acetylate proteins at physiological levels *in vivo* (Wagner and Payne, 2013). Furthermore, attempts to reduce the levels of AcCoA, such as by draining acetyl groups into an acetyl group polymer such as polyhydroxybutyrate, do not avoid any contribution by KATs (Hu, 2013). Work in an organism where AcCoA can be non-essential, such as the reduced genome bacterium *Borrelia burgdorferi* that solely synthesizes AcCoA to generate mevalonate, a precursor of cell wall lipid I, might be a useful reductionist system to determine whether this truly occurs *in vivo* (Bontemps-Gallo et al., 2018). Without knowing all possible KATs and eliminating them, it is hard to truly ever say whether an AcCoA-dependent acetylation is truly non-enzymatic *in vivo*.

**FIGURE 2:**
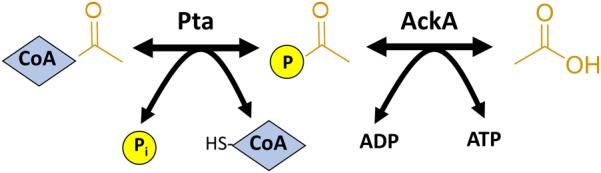
The Pta-AckA acetate fermentation pathway generates AcP as an intermediate. Phosphotransacetylase (Pta) converts AcCoA to AcP by substituting CoA for inorganic phosphate (P_i_). Acetate kinase (AckA) converts AcP to acetate, generating an ATP in the process. This pathway is reversible.

Fortunately, modulation or complete elimination of AcP levels is much simpler and less detrimental to many bacteria, which greatly facilitates studying AcP-dependent acetylation. In *E. coli* and many other bacteria, AcP is generated as an intermediate of the acetate fermentation pathway catalyzed by the reversible enzymes acetate kinase (AckA) and phosphotransacetylase (Pta) (Rose et al., 1954). Pta catalyzes the conversion of AcCoA and inorganic phosphate to AcP and free CoA. AckA then converts AcP into acetate by transferring the phosphoryl group to ADP to generate one molecule of ATP (Figure 2). In the presence of high acetate concentrations (>10 mM), the pathway can run in reverse, synthesizing AcCoA from acetate, ATP, and CoA (Brown et al., 1977; Kumari et al., 1995). By genetically manipulating this pathway, the levels of AcP and thus acetylation can be altered. For example, if cells are grown on glucose, the cells ferment acetate and generate AcP as an intermediate. If one prevents the conversion of AcCoA to AcP by deleting Pta or the entire Pta AckA pathway, AcP cannot be generated. When these mutants are analyzed by western blot, the intensity of the acetylated proteins is greatly reduced to background levels (Weinert et al., 2013a; Kuhn et al., 2014). It is worth noting that acetylation can still be detected by mass spectrometry in Δ*pta* and Δ*pta ackA* mutants, but the relative amount of acetylation and number of acetylated lysines is greatly reduced. On the other hand, by deleting *ackA*, AcP accumulates to 10–20 mM (Klein et al., 2007; Wolfe, 2010; Weinert et al., 2013a), which can be seen via western blot as a greatly intensified acetylation profile relative to wild-type cells (Weinert et al., 2013a; Kuhn et al., 2014). Mass spectrometry confirms that many more unique lysines on many more unique proteins become acetylated in this mutant, and those lysines that are detected as acetylated in wild-type become further acetylated as the proportion of the total pool of each unique peptide shifts toward acetylated (Weinert et al., 2013a; Kuhn et al., 2014).

## Targets of Non-Enzymatic, AcP-Dependent Acetylation

To date, only a handful of groups have tried to directly determine the impact of acetylation by AcP on the acetylome of an organism (Weinert et al., 2013a, 2017; Kuhn et al., 2014; Post et al., 2017; Bontemps-Gallo et al., 2018; Reverdy et al., 2018). This has been achieved by mass spectrometry analysis of wild-type cells and comparing the acetylated proteins to acetylated proteins cells where the levels of AcP have been manipulated, usually by deleting *ackA* to accumulate AcP in cells or by deleting *pta* to prevent synthesis of AcP. Due to the prevalence of AcP-dependent acetylation, especially under conditions when cells are exposed to high carbon concentrations, much of the acetylation detected in wild type bacteria is likely a result of acetylation by AcP (Schilling et al., 2015).

These studies result in large data sets with hundreds of lysines acetylated from multiple organisms. Often, to get a picture of what processes these acetylated lysines may be involved in, the modified proteins can be grouped by keyword enrichment, gene ontology term enrichment, and Kegg pathway enrichment. Across these studies and grouping strategies, the modified proteins fall into multiple processes, but the two most common are translation and central metabolism.

From the current *E. coli* data sets, it appears that most of the ribosomal subunits, most of the aminoacyl-tRNA ligases, and all of the elongation factors are acetylated on multiple lysines in an AcP-dependent manner. In contrast, AcP acetylates only one lysine on one initiation factor (Kuhn et al., 2014; Schilling et al., 2015). Whether any of these acetylations impact the rate or fidelity of translation has yet to be determined. However, it has been shown that charging of tRNAs can be inhibited by AcP-dependent acetylation of certain aminoacyl-tRNA ligases (Venkat et al., 2017a; Ye et al., 2017).

Similarly, most central metabolic enzymes are acetylated in an AcP-dependent manner; this is true for all of the glycolytic pathways of *E. coli* and *B. subtilis* (i.e., Embden–Meyerhof–Parnas, Pentose Phosphate, and Entner–Doudoroff). Currently, the functional consequences of most of these acetylations remain largely undetermined. However, it is reported that AcP-dependent acetylation abolishes the activity of the glycolytic enzyme enolase from both *E. coli* and *B. subtilis* (Nakayasu et al., 2017) and inhibits the activity of glyceraldehyde 3-phosphate dehydrogenase A from *Borrelia burgdorferi* (Bontemps-Gallo et al., 2018). The TCA cycle is also highly acetylated by AcP. Thus far, malate dehydrogenase is the rare example of a central metabolic enzyme whose activity is enhanced by AcP-dependent acetylation (Venkat et al., 2017b).

## Structural Determinants of Non-Enzymatic Acetylation

**FIGURE 3:**
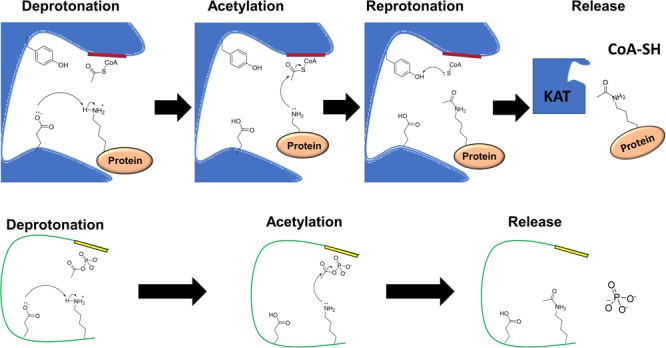
Catalytic mechanisms of enzymatic and non-enzymatic acetylation. **(Top)** In the enzymatic mechanism, a lysine to be acetylated binds at the acceptor site of a lysine acetyltransferase (KAT) and AcCoA binds at the donor site of the KAT called the P-loop (red) with consensus motif Gln/Arg-x-x-Gly-x-Gly/Ala (Salah Ud-Din et al., 2016). A catalytic glutamate deprotonates the epsilon amino group of the target lysine. The lysine performs a nucleophilic attack on the carbonyl carbon of AcCoA, resulting in acetylation of the lysine. The CoA group becomes protonated by a tyrosine, which regenerates the KAT and facilitates the release of the free CoA and the target protein. **(Bottom)** In this example of non-enzymatic acetylation, AcP is bound through its negatively charged phosphoryl group to a neighboring patch (yellow) that contains positively charged residues and/or residues that can form hydrogen bonds. In this example, the lysine is deprotonated by a glutamate on the same protein. The lysine performs a nucleophilic attack on the carbonyl carbon of AcP resulting in an acetyllysine. Inorganic phosphate is released as a byproduct of the reaction. A mechanism similar to this can be considered for non-enzymatic AcCoA-dependent acetylation.

While non-enzymatic acetylation does not require a KAT, analysis of the environments that contain acetylated lysines suggest that the mechanism by which this enzyme-independent reaction occurs has many parallels with enzymatic acetylation (Figure 3). While the KAT serves as an AcCoA-binding protein to properly position the acetyl group near the target lysine, non-enzymatic acetylation requires that the protein being acetylated coordinates the CoA group for AcCoA or the phosphate moiety for AcP (Kuhn et al., 2014). Often, the coordinating amino acids are positively charged (Arg and Lys), which make ionic bonds, or polar amino acids that make hydrogen bonds via hydroxyl groups (Ser, Thr, and Tyr) or side chain amides (Gln and Asn) (Kuhn et al., 2014). In contrast to KAT-dependent acetylation, where a glutamate from the KAT typically extracts a proton from the lysine to be acetylated, during non-enzymatic acetylation, the non-enzymatic process requires an internal glutamate or a water molecule (Okanishi et al., 2013; Kuhn et al., 2014). It may be expected that lysines acetylated non-enzymatically may have a reduced pK_a_, and thus more propensity to lose a proton. However, the pK_a_ has not been found to correlate with reactivity (Baeza et al., 2015). Like KATs and KDACs, there is no clear motif that is predictive of whether a lysine is susceptible to chemical acetylation. This was shown by using AcP to acetylate a peptide library with the sequence GXKZGC, where X and Z are each a standard amino acid with the exception of cysteine, on a SAMDI chip. This method showed that almost every peptide could be acetylated, but selectivity and specificity could be achieved by adding magnesium and salt (Kuhn et al., 2014). The SAMDI results indicated adjacent arginines and lysines favored acetylation; *in vivo*, however, there is a propensity for glutamates and aspartates to be adjacent to the lysines acetylated by AcP. Similar results were found for AcCoA, where glutamates were found near lysines *in vivo* (Baeza et al., 2015). For both AcP and AcCoA, there is a tendency for susceptible lysines to be surface-exposed (Kuhn et al., 2014; Baeza et al., 2015). However, due to their relatively small size, AcP and AcCoA can access buried lysines that a KAT could not (Kuhn et al., 2014). Thus, protein structures could have evolved to utilize non-enzymatic acetylation for roles that are impossible for enzymatic acetylation.

## Regulation of AcP-Dependent Acetylation – a Consequence of Fermentation

**FIGURE 4:**
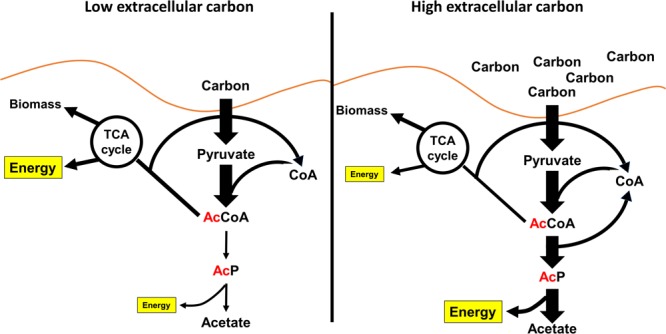
AcP is made as a consequence of overflow metabolism. **(Left)** When bacteria like *E. coli* are growing on low concentrations of carbon, much of the carbon is put into the TCA cycle, lipid metabolism, and metabolite biosynthesis. Acetate production is very low. **(Right)** When the concentration of carbon increases, the flux of carbon through glycolysis depletes the limiting pool of free CoA. To continue to consume carbon, *E. coli* can regenerate free CoA by fermenting acetate. This results in the production of AcP.

When the glucose consumption rate depletes free CoA faster than it can be recycled, *E. coli* will ferment acetate from AcCoA via the Pta-AckA pathway (Figure 4). This occurs even in the presence of oxygen in a process called overflow metabolism, akin to a safety valve that regenerates the limiting pools of CoA (Hollywood and Doelle, 1976; Andersen and von Meyenburg, 1980). However, the production of AcP by Pta is faster than the conversion of AcP to acetate by AckA, leaving a pool of the intermediate AcP (Klein et al., 2007; Keating et al., 2008). Thus, when *E. coli* cells are grown in the presence of excess carbon such as glucose, the bacteria ferment acetate and produce AcP. Thus, ameliorating the need for cells to ferment reduces acetylation. Such an example is when cells lacking the major glucose transporter EIICB^glc^ are grown in medium supplemented with glucose. These mutants must assimilate glucose through alternative glucose transporters such as the galactose permease GalP. The use of these alternative transporters reduces the rate of glucose flux into the cell. As such, the cells also produce less acetate and, as expected, have reduced acetylation (Steinsiek and Bettenbrock, 2012; Schilling et al., 2015).

This phenomenon is not exclusive to glucose. Lactate, fructose, and xylose can also potently induce acetylation in *E. coli* as assessed by anti-acetyllysine western blot (Schilling et al., 2015, 2019; Christensen, 2019). Mass spectrometric analysis has determined the acetylomes of cells grown in xylose, a pentose sugar, and cells grown in glucose, a hexose sugar (Schilling et al., 2019). Glucose and xylose are catabolized through different metabolic pathways; however, each pathway results in acetate fermentation. As such, it may not be surprising that, regardless of carbon source, almost every acetylated lysine was similarly regulated in glucose or xylose. The one exception was xylose isomerase, a protein necessary for catabolism of xylose, which had two lysines differentially acetylated in glucose and xylose. These results suggest that cells regulate AcP-dependent acetylation simply by regulating overflow metabolism and production of AcP.

One other key physiological shift that causes accumulation of acetylations is cessation of growth, such as entry into stationary phase. For both *E. coli* and *N. gonorrhoeae*, acetylation increased as the culture entered stationary phase either naturally or when artificially induced (Weinert et al., 2013a; Kuhn et al., 2014; Schilling et al., 2015; Post et al., 2017). We recently determined why acetylation accumulates after *E. coli* cultures enter into stationary phase. *E. coli* cultures grown in glucose-supplemented peptide-based media consume both amino acids and glucose during exponential growth; however, most of the glucose is consumed after the culture enters into stationary phase (Christensen et al., 2017).

Compared to exponential phase, two facets of stationary phase make it the perfect growth phase to promote acetylation when excess carbon is present: reduced protein synthesis and reduced respiration. In stationary phase, rates of growth cannot be sustained due to depletion of a limiting nutrient (Reeve et al., 1984). Thus, cells reduce rates of transcription and translation, which greatly reduces the generation of new proteins (Navarro Llorens et al., 2010). Since the rate of protein biosynthesis is reduced, fewer TCA cycle intermediates are used to generate new biomass. This will cause a bottleneck at the TCA cycle, and carbon must enter other pathways. In conjunction with reduced translation, total cellular protein becomes further reduced due to increased protein turnover. When *E. coli* enters stationary phase, aerobic metabolism is repressed (Nyström, 2004; Navarro Llorens et al., 2010). As such, the carbon flux is driven toward fermentation. The combination of a reduced proteome in stationary phase and the continued metabolism of a fermentable carbon source increase the probability that any susceptible lysine will become acetylated.

In conclusion, data thus far suggests that non-enzymatic AcP-dependent acetylation is an unavoidable consequence of fermentation that accumulates on an aging proteome. Since AcP is made as an intermediate of fermentation, the only means of regulating acetylation would be by regulating fermentation, deacetylation of acetyllysines with a KDAC, and/or evolving protein structures to optimally favor/disfavor acetylation of specific lysines. Because acetate fermentation has evolved as a key mechanism that allows many bacteria to regenerate the limited pool of CoA, this would suggest that *E. coli* has evolved over millions of years to cope with or utilize this global modification.

## A Possible Evolutionary History of Acetylation and Because of Acetylation

Because nearly all life has evolved to utilize activated acetate as a key molecule in metabolism, all organisms must inevitably cope with the potential for protein acetylation. To understand the physiological relevance of acetylation, it is helpful to first imagine how acetylation evolved. As proposed previously, AcP is a relatively simple molecule compared to ATP or AcCoA with higher energy, and as such is likely to be a more ancient energy currency (Ninfa et al., 2000; Wolfe, 2010). Due to the well-understood effects of phosphorylation by AcP, it was proposed that proteins like response regulators could have evolved to accept phosphoryl groups from AcP, and only later would the cell have evolved phosphatases to remove those phosphoryl groups. Finally, some phosphatases would evolve to become kinases to fine-tune these pathways (Wolfe, 2010).

Similarly, the ability for AcP to acetylate proteins also could be a driver of evolution. Acetylation of a catalytic lysine can be extremely detrimental to protein function, and inability to remove these acetylations could result in a dead enzyme. Thus, cells must have evolved deacetylases to ensure that a given lysine would be unmodified despite the presence of endogenously produced reactive metabolites. Contrary to the phosphorylation example, the acetyltransferases are not homologous to the deacetylases and must have evolved independently. Perhaps these acetyltransferases evolved after the development of their primary substrate, AcCoA.

Each acetylated lysine has the potential to be regulatory. Whether these acetylations are due to non-enzymatic mechanisms or catalyzed enzymatically, life must balance the possibility of detrimental acetylation with benign or beneficial acetylations. The best ways to ensure that only optimal lysines are acetylated under a given condition would be to either (1) evolve a lysine deacetylase to remove deleterious modifications or (2) evolve proteins such that they do not have critical lysines susceptible to acetylation. For *E. coli*, almost all acetylated linear peptides are susceptible to CobB (AbouElfetouh et al., 2015), but not all acetylated *E. coli* proteins are CobB-sensitive *in vivo* (Kuhn et al., 2014). The CobB-sensitive lysines tend to be surface-exposed (AbouElfetouh et al., 2015). Indeed, for a lysine to be deacetylated, it almost certainly cannot be buried within the protein structure, unless the acetylated residue becomes accessible through “protein breathing,” large scale conformational changes of the protein structure (Kossiakoff, 1986). However, the extent of breathing *in vivo* is probably restricted due to molecular crowding in the cytoplasm (Makowski et al., 2008). Thus, the protein must be structured in such a way to favor deacetylation of the target lysine without disrupting the activity of the protein.

If an acetylated lysine blocks protein function and is not accessible to CobB or another deacetylase, the cell must replace that protein by degrading it and synthesizing an entirely new protein. Not all lysines can be acetylated, but those that do become modified can be acetylated at different rates (Baeza et al., 2015; Schilling et al., 2015). As buried lysines would be refractory to CobB, one would also expect that acetylation of these lysines is rarely enzymatic due to the inability for an enzyme to access the lysine. Thus, evolution may have selected for molecular environments that disfavor acetylation of these buried and other critical lysines. However, any changes made over evolution that would disfavor acetylation had to be balanced with a protein sequence that permits proper folding and activity.

## Future Thoughts

The study of acetylation is a fast growing field with technological advances in analytical methodologies, such as mass spectrometry, providing a greater depth of analysis. The report of each new acetylome opens more avenues to explore. While some acetylations are certain to be organism-specific, there are bound to be many conserved acetyllysines in critical processes, as we have already observed in central metabolism and translation. Each of these acetyllysines must result from one or more of the recognized acetylation mechanisms, either enzymatic or non-enzymatic. In turn, these mechanisms may themselves be regulated dynamically with the potential for signaling cascades akin to phosphorylation.

Researchers in this new field have two main routes that they can follow. They can investigate the effect of acetylation on individual sites on individual proteins or they can study the effect of global acetylation. The former route can take advantage of the numerous acetylome studies that have yielded data sets containing hundreds of acetylated proteins modified on thousands of unique lysines. The problem is how to prioritize the most functionally relevant acetylation sites. Typically, researchers prioritize by choosing lysines in provocative locations on proteins with easily assessed phenotypes or activities. However, it should be noted that genetic mimic/genetic ablative mutants are often used to compare extreme stoichiometries, i.e., a mimic of the 100% acetylated state versus a mimic of the 100% unacetylated state. The same is true of proteins acetylated by non-canonical amino acid substitution.

Another way to prioritize is to determine the stoichiometry of each acetylated lysine (Baeza et al., 2014; Nakayasu et al., 2014; Weinert et al., 2015, 2017; Meyer et al., 2016; Miyagi, 2017; Wei et al., 2018). A lysine with a large fold change but a low basal stoichiometry may be a less physiologically important acetylation event compared to one with a smaller fold change but a higher basal stoichiometry. Unfortunately, obtaining the stoichiometry of the acetylome is a non-trivial technical challenge for all but a few groups. To make matters worse, most of the existing analyses were performed on highly mutagenized laboratory strains grown under conditions that make physiologically relevant interpretations difficult.

Whereas site-specific or protein-specific investigations can provide lessons concerning the manner in which acetylation influences protein structure/function relationships, such focused studies cannot explain the global effect of simultaneous low stoichiometric acetylation of large numbers of lysines, as occurs by non-enzymatic acetylation. These acetylations greatly expand the acetylome, neutralizing thousands of positive charges, yet the impact of these modifications remains unclear. Furthermore, acetylation is not the only global modification. The proteome can be acylated using many other central metabolites that may compete for the same lysines (Peng et al., 2011; Zhang et al., 2011; Colak et al., 2013; Wagner and Payne, 2013; Weinert et al., 2013b; Kosono et al., 2015; Singhal et al., 2015; Yang et al., 2015; Meyer et al., 2016; Mizuno et al., 2016; Sun et al., 2016b; Basisty et al., 2018; Gaviard et al., 2018; Vasileva et al., 2018; Wei et al., 2018). The potential crosstalk amongst these acylations and between these acylations and other PTMs presents an important challenge as we attempt to truly understand the inner workings of the cell.

Since lysines can be acylated without the use of an enzyme, we would suspect that bacteria would have evolved to utilize this otherwise “wasteful” process. We propose that global acylation may serve many functions. For example, acylations may serve as a feedback mechanism to slow an excessive carbon flux. Acylations could protect proteins against damaging modifications, such as carbonylation, that result in protein misfolding and aggregation (Dalle-Donne et al., 2003; Maisonneuve et al., 2008). Acylations may be less severe modifications that could protect proteins against such harmful lesions. It may also be possible for acetylation to serve as a method for cells to store two carbon subunits. While there are designated carbon storage polymers, such as starch in plants or glycogen in animals, fungi, and bacteria, perhaps acetylation is a primordial form of carbon storage.

Finally, researchers should remember that heterologous expression of a protein in *E. coli* or any other bacterium carries the possibility that the purified protein preparation may be a heterogeneous mixture decorated by various acylations. Indeed, at least one group has reported that recombinant insulin produced in *E. coli* is acetylated (Szewczak et al., 2015). The potential for acylations to influence efficacy has great implications for the pharmaceutical industry, but also for any researcher who uses a heterologous system to express their protein of interest. Depending on the protein and the conditions under which the host bacteria are grown, the modified protein may behave in a manner that is not consistent with expectations.

## Author Contributions

DC, BS, and AW outlined the review. DC wrote the first draft and designed the figures. XX, NB, JB, and SM helped to complete the review, especially sections focused on structural biology and mass spectrometry. AW did the final edit. BS and AW oversaw the entire process of this study.

## Conflict of Interest Statement

The authors declare that the research was conducted in the absence of any commercial or financial relationships that could be construed as a potential conflict of interest.
